# News Items

**Published:** 1872-04-15

**Authors:** 


### News Items.

The Medical Profession in Russia.—From official papers it appears that during the past year 10,000 medical men carried on practice. Of these about 6,113 were in the service of the Government, and 4,686 independent of it. These figures give one medical man for 7,182 inhabitants. In many districts a long journey must be performed before a medical man is found. In the United States of America there are 49,798 doctors for the thirty-nine millions of inhabitants—viz., one medical man for every thousand inhabitants.—*Lancet, April*, 1872.

Health Officers' Report for Philadelphia for 1871.—The births were 18,346; marriages 6,806; deaths 16,993. The principal epidemic was small-pox.

In the early part of the year this loathsome disease made its appearance, but did not attract any particular attention until August, when it began to assume the form of an epidemic, and continued gradually until the month of October, when we registered three hundred and thirty-one (331) deaths; it still continued with greater violence until the end of the year, when our entire mortality reached one thousand eight hundred and seventy-nine (1,879); previous to October, we only had registered forty-seven (47) deaths, thus leaving one thousand eight hundred and thirty-two (1,832) in the months of October, November, and December.

Scarlet fever shows 262 deaths; cholera infantum, 829; consumption, 2,237; and croup, 264. Of the total, 27 per cent. were children under one year of age, and 46 per cent. children under ten. The rates of deaths to population was one in forty-four; the highest in any one ward, one in twenty-nine. —*Philadelphia Med. and Surg. Reporter.*

Chronology of American Medical Journalism.—Prof. Noble Young, M.D., of the Georgetown Medical College, D. C., said, in his address on the laying of the corner-stone of the building for the College of Physicians and Surgeons, Wilmington, N. C.:

"The honor of establishing the first medical journal in the United States is due to New York by the labors of Doctors Samuel M. Mitchell, Edward Miller, and Elihu H. Smith, in 1797. The *Philadelphia Medical and Physical Journal* was next published in 1804, followed by the *Philadelphia Medical Museum*, in 1805; *Baltimore Medical and Physical Recorder*, 1808; *New York Medical and Philosophical Journal and Review*, in 1809; *The American Medical and Philosophical Register*, in New York, in 1810; *The American Mineralogical Journal*, at New York, in 1810; *Eclectic Repository*, in Philadelphia, in 1811; *Baltimore Medical and Philosophical Lyceum*, in 1811; *New England Journal of Medicine and Surgery*, Boston, in 1812; *American Medical Recorder*, Philadelphia, in 1818; *Philadelphia Journal of Medicine and Physical Sciences*, in 1820; *American Journal of Science and Arts*, New Haven, in 1821; *New York Medical and Physical Journal*, 1822; *Western Medical Reporter*, Cincinnati, Ohio, 1822; *Hartford Analectic Journal of Medicine and Surgery*, 1823; *Boston Medical Intelligencer*, 1823; *Medical Review and Analectic Journal*, Philadelphia, 1824; *New York Monthly Chronicle of Medicine and Surgery*, 1824; *Carolina Journal of Medicine, Science and Agriculture*, at Charleston, S. C., in 1825."—*Philadelphia Med. and Surg. Reporter.*

The University of Strasbourg is to commence operations under German auspices May 1st. The Medical Faculty are, Dr. Waldeyer, Anatomy; Dr. Hoope Seyler, Physiology; Von Recklinghausen, Pathological Anatomy; Dr. Schniedeberg, Materia Medica; Dr. Leyden, Internal Pathology; Dr. Lucke, Dr. Gussbrow, Gynæcology and Obstetrics. This is a fine array of talent.

Falling off at Harvard.—Since the adoption of the extended curriculum at Harvard, the number of students has diminished by 105, which shows precisely the number of students attending lectures there whose only desire is to get a diploma with as little trouble as possible.

A Heavily Backed Journal.—A new Journal of Ophthalmology has been started in Paris. Its cover bears the names and titles in full of two directors, fifteen editors in chief, including the directors, and thirteen corresponding editors.

Privy Councillor Traube, of Berlin, has been appointed to the position of professor-in-ordinary at the University of Berlin. Professor Traube is the first individual of Jewish faith ever appointed to this position in Berlin.

Varicella in San Francisco.—Chicken pox has been prevailing lately in San Francisco in an aggravated form, some cases so near to small pox in character as to have been reported as such to the Board of Health.

To Disguise Castor Oil.—Rub up two drops oil of cinnamon with an ounce of glycerine, and add an ounce of castor oil. Children will take it as a luxury and ask for more.

